# A New Cd(II)-Based Coordination Polymer for Efficient Photocatalytic Removal of Organic Dyes

**DOI:** 10.3390/molecules28196848

**Published:** 2023-09-28

**Authors:** Juanjuan Zhao, Zhuoyu Dang, Mohd. Muddassir, Saleem Raza, Aiguo Zhong, Xiaoxiong Wang, Juncheng Jin

**Affiliations:** 1 School of Physics and Materials Engineering, Hefei Normal University, Hefei 230601, China; 2 Department of Chemistry, College of Sciences, King Saud University, Riyadh 11451, Saudi Arabia; muddassir@ksu.edu.sa; 3 College of Chemistry and Life Sciences, Zhejiang Normal University, Jinhua 321004, China; 4 School of Pharmaceutical and Chemical Engineering, Taizhou University, Taizhou 318000, China; zhongaiguo@tzc.edu.cn; 5 School of Materials and Environmental Engineering, Shenzhen Polytechnic University, Shenzhen 518055, China; wangxiaoxiong20@szpt.edu.cn; 6 Key Laboratory of Biomimetic Sensor and Detecting Technology of Anhui Province, West Anhui University, Liuan 237012, China

**Keywords:** coordination polymer, cadmium ions, mechanism, dyes degradation

## Abstract

Coordination polymers (CPs) are a diverse class of multi-dimensional compounds that show promise as photocatalysts for degrading dyes in polluted water. Herein, a new 1D Cd(II)-based coordination polymer with the formula [Cd(bpyp)(nba)_2_] (**1**) (bpyp = 2,5-bis(pyrid-4-yl)pyridine and Hnba = 4-nitrobenzoic acid) is synthesized and characterized. In **1**, the two carboxyl groups of two different nba^−^ ligands show *μ*_2_-η^1^:η^1^ and *μ*_1_-η^1^:η^1^ coordination modes to connect the Cd^II^ centers and sit on either side of the chain along the *b* direction. The produced CP **1** was utilized as the photocatalyst in the process of the photodegradation of methyl blue (MB), methyl orange (MO), rhodamine B (RhB), and methyl violet (MV) dyes when exposed to UV light. The photocatalytic degradation activities of CP **1** were analyzed, and the results suggest that it exhibits an extraordinary efficiency in the degradation of MB, MV, MO, and RhB. RhB has a 95.52% efficiency of degradation, whereas MV has a 58.92% efficiency, MO has 35.44%, and MB has 29.24%. The photodecomposition of dyes is catalyzed mostly by •O_2_^−^ and •OH^−^, as shown by research involving the trapping of radicals.

## 1. Introduction

The extreme toxicity and non-degradability of persistent hazardous chemicals make water pollution a severe threat to modern human health and ecology [1,2]. The most dangerous of these chemicals include heavy metals, artificial colors, and organic micro-pollutants. Water contamination issues have become more severe as industrialization progresses [3]. Toxic wastewater, such as that generated by printing, dyeing, textiles, and other industries, may pollute underground and aboveground water supplies [4,5]. Methylene blue (MB) and rhodamine B (RhB) are two organic dyes with a wide variety of industrial applications; MB is even considered a pharmaceutical due to its widespread presence in these fields. They are crucial in several manufacturing processes [6,7]. This explains why MB and RhB are found in wastewater treatment plants. Although MB and RHB may worsen environmental conditions, they also represent a health hazard and potentially cause cancer in humans [8]. These contaminants are very persistent, sometimes remaining in water for decades. Adsorption and biological sewage treatment procedures have limited success in removing them, and they may even contribute to secondary contamination if not used with caution [9,10]. These long-lasting poisons may lead to cancer, mutations, deformities, and severe organ and immune system damage. As a direct consequence of this, the identification and/or elimination of these poisons in the environment must be carried out promptly and effectively. Adsorption is one method that has been used in removing hazardous compounds and/or detecting their presence [11], biodegradation, ozonation, and membrane filtration [12,13]. Although the absorption method is straightforward, it has drawbacks, such as poor selectivity and sluggish process kinetics, which must be addressed [14]. An appropriate and reusable technique that has recently been proposed is photocatalytic degradation. Solid metal oxides and sulfide semiconductor photocatalysts, such as titanium dioxide, zinc oxide, and metal sulfides, are often utilized [15]. However, each has unique drawbacks, such as a limited spectrum response range and poor quantum conversion efficiency. TiO_2_ has little reaction to spectra since it is only active in UV light, which equates to about 4–6% of visible light [16,17,18]. Electrochemical reactions may lead to photo corrosion; thus, it is essential to find ways to increase efficiency and durability [19,20].

The development of new photocatalytic materials with high quantum efficiency and outstanding visible light responsiveness is thus crucial. Coordination polymers with regular crystal structures have lower chances of electron-hole recombination [21,22,23]. The large inner surface area, tunable inner pore size, and high porosity not only pre-concentrate low-concentration samples but also provide additional reaction sites, increasing the photoreactivity of the materials [24,25]. Additionally, metal centers and organic ligands may successfully control their band gap and photocatalytic activity. Based on the benefits mentioned earlier, multifunctional materials have drawn a lot of attention recently as society has advanced and the need for smart materials has increased [26]. A potential inorganic–organic hybrid crystalline material with applications in adsorption is coordination polymers (CPs), which have rich and diverse architectural compositions, catalysis, sensing and detection, gas capture, and drug delivery [27,28,29,30,31,32,33]. Several desirable characteristics make CPs promising adsorbents, including having a high surface area and pore volume, high chemical stability, tunable properties, and topologies capable of strong hydrogen bonding and electrostatic interactions with the target adsorbate. Additionally, their good adsorption efficiency, good selectivity for the target molecule, and acceptable reusability for several adsorption cycles are all characteristics that make CPs cost-effective [34,35]. Multiple studies have shown the efficacy of coordination polymers as photocatalysts for eliminating hazardous substances in the natural environment [36].

In addition to having the capacity to act as a photocatalyst, CPs may also display remarkable luminous capabilities. It is well known that d^10^-transition metal ions, such as Zn^2+^ and Cd^2+^, have increased emissions due to MLCT (metal-to-ligand charge transfer) [37]. As a result, these ions are commonly chosen in the luminous coordination polymer production process. The luminescent coordination polymers (LCPs), thanks to their advantageous properties, such as their simplicity of production, rapid response, and high sensitivity, are well suited for detecting environmental toxins at trace concentrations [38,39]. Herein, we synthesized a new cadmium-based coordination polymer **1**, then characterized and investigated its photocatalytic activity against different dyes.

## 2. Results and Discussion

### 2.1. Structural Discussion of [Cd(bpyp)(nba)*_2_*] (***1***)

**Figure 1:**
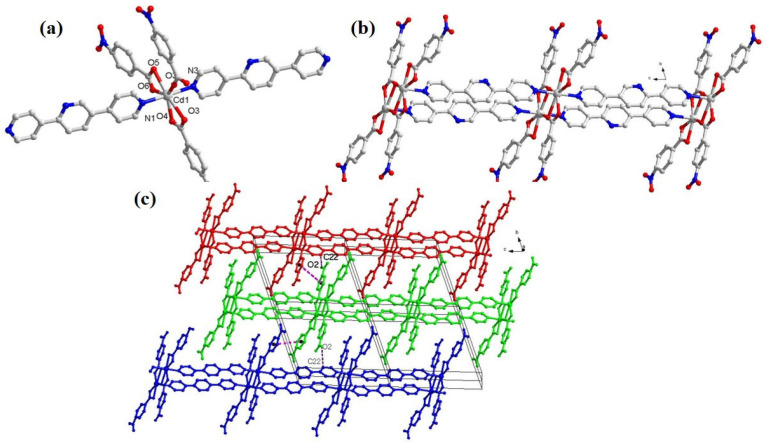
(**a**) View of the coordination geometry of Cd(II) center; (**b**) the double-chain connected by bpyp and nba linkers; (**c**) 3D supramolecular network connected by hydrogen bonds and packing interactions.

The asymmetric unit of **1** has a Cd(II) center, two nba ions, and one 2,5-bis(pyrid-4-yl)pyridine linker. Two types of coordination fashions of the nba anion are exhibited in the asymmetric unit (Scheme S1); the carboxylate group of one linker shows a bidentate chelate mode coordinating to one Cd ion while the other anion shows a bidentate bridging mode connecting two Cd(II) ions. The Cd(II) ion has a seven-coordinate geometry defined by five oxygen atoms from three NO_2_-nba linkers and two nitrogen atoms from two bpyp to form a trigonal bipyramid geometry. The bond distances of Cd–O fall in the 2.306 (2)–2.443 (2) Å range, and Cd–N bond length falls between 2.294 (3) and 2.310 (3) Å, which is in the normal range. The coordination angles around the Cd atom are in the range of 54.28 (8)–173.78 (10)° (Table S2). The cisoid O–Cd–O and transoid N–Cd–Obond angles fall in the region of 54.28 (8)–144.41 (9)° and 86.28 (9)–99.13 (9)° for **1**, thus displaying normal bond lengths and angles [40,41,42]. Compared to Cd-atoms in **1**, the cisoid O–Cd–O and transoid N–Cd–O bond angles of Mn (II)-based CPs exhibit a more significant departure from the corresponding value for a regular octahedron (theoretical value: 90° and 180°) [43], suggesting the center Cd-atom of **1** has a significantly less distorted geometry. The above conclusion may be due to the electron drawing effect of the nitro group, which may strengthen the coordination geometry. Two carboxylate groups connect two symmetry-related metal atoms’ Cd centers to shape eight-membered rings. In **1**, the two carboxyl groups of two different nba^−^ ligands show *μ*_2_-η^1^:η^1^ and *μ*_1_-η^1^:η^1^ coordination modes to connect the Cd^II^ centers and sit on either side of the chain along the *b* direction (Scheme S1). The dinuclear units are pillared through bpyp bridging to generate a double chain (Figure 1b). The carbon atoms donate one hydrogen to form inter-chain hydrogen bonds to the carboxylate oxygen atom with a distance of C22–H22···O2 = 3.522 Å, respectively, including a 2D network (Figure 1c). Finally, **1** is extended into a 3D supramolecular network through the offset face-to-face π–π stacking interactions in which the mean interplanar distance between adjacent carboxylate ligands belonging to neighboring layers is 3.715 Å (Figure 1c) [44,45].

### 2.2. Thermogravimetric Analysis and SEM

The polymeric complexes are determined through a thermogravimetric analysis (TGA) conducted within a nitrogen environment, with a temperature range from 40 to 800 degrees Celsius. The graphical representation in Figure S2 illustrates that **1**’s degradation predominantly occurs through two distinct processes. The thermal stability of the first critical point of CP **1** is 158 °C. The thermal decomposition of the complex’s nba and polymeric chain resulted in a gradual weight loss, with an isothermic peak observed at 350 °C and 750 °C during the first 25% and subsequent 70% mass loss, respectively. The entire complex underwent a slow weight loss as it collapsed. Our SEM images of **1** indicate a cubic/rectangle shape with an irregular size (Figure S3).

### 2.3. Optical Property

Tauc’s formula was used to calculate the energy of the optical band gap and determine the conductivity of the complex. Tauc’s formula is shown in Equation (1).

*(αhυ)^n^ = A(hυ* − *E_g_)* (1)

where *α* denotes the absorption coefficient, and it is calculated by *α* = (2.303 × absorption)/*t*, *t* = cube thickness (1 cm). *A* refers to a constant, *E_g_* indicates the band gap energy, the exponent *n* depends on the type of transition, and *h* denotes the Planck’s constant. Equation (1) was used to plot *(αhυ)*^2^ vs. *hυ* (Figure S4), which calculated the band gap energy of the complex by extrapolating its linear portion. The optical band gap energy of **1** is 2.85 eV. The optical band gap energy suggested that **1** is semiconducting. Therefore, we further investigated its photocatalytic activity against pollutant dyes.

### 2.4. Photocatalytic Study

**Figure 2:**
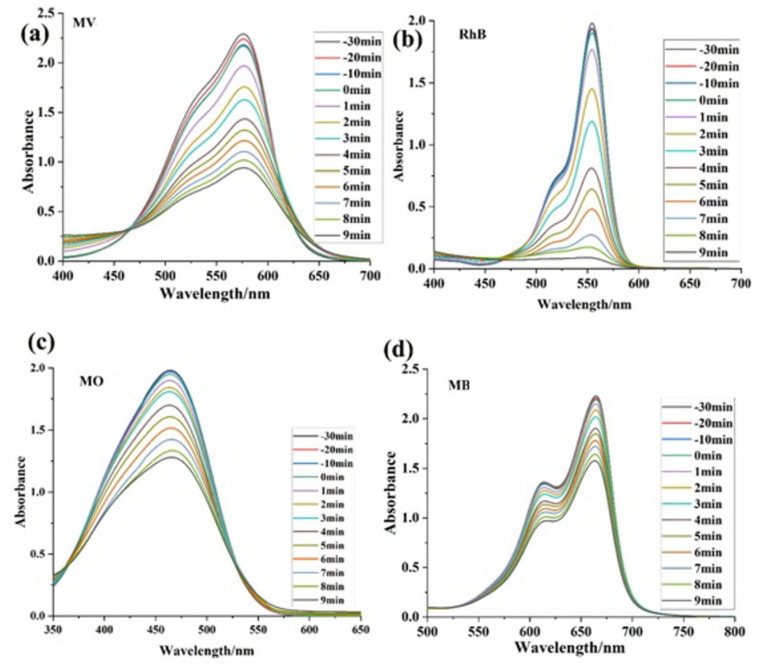
Dye degradation spectra of (**a**) Methyl Violet (MV), (**b**) Rhodamine-B (RhB), (**c**) Methyl orange (MO), and (**d**) Methylene blue (MB).

To examine the catalytic efficacy of the photocatalyst acquired, a range of experiments were carried out to assess the degradation of MO, MV, MB, and Rh B by **1**, while being exposed to visible light at ambient temperature. The outcomes of these experiments are depicted in Figure 2. Before the photocatalytic experiment, the system underwent a 30 min equilibration period in the absence of light. The photocatalysis process involves a crucial step of the dyes’ adsorption on the catalyst’s surface [46]. The preliminary stage before dye degradation typically involves the satisfactory adsorption of dye molecules. The adsorption of dye molecules onto the surface of the catalyst significantly impacts the light utilization rate of photocatalyst **1** and the production of active free radicals, which are crucial factors in determining the rate of photodegradation. It is widely recognized that dye molecules exhibit a remarkable stability when exposed to visible light, resulting in minimal self-photodegradation. The results depicted in Figure 2a–d indicate that a range of dyes underwent varying degrees of degradation within 9 min.

**Figure 3:**
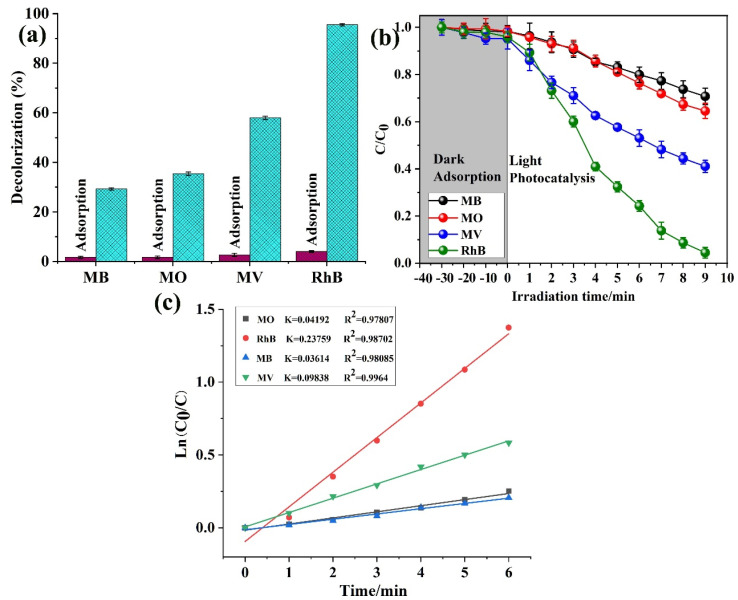
(**a**) Adsorption value before the degradation (the blue color is degradation efficiency); (**b**) degradation study of all four dyes; and (**c**) kinetic study of all four types of dyes.

Furthermore, the adsorption and decolorization of all these four dyes were investigated as shown in Figure 3a. According to the result, the adsorption ability of **1** toward the RhB dye is very high, while the other is not very significant as compared to that of RhB. Furthermore, the adsorption study was checked with dark and light conditions, and the degradation rate of RhB is higher than the other three dyes, as shown in Figure 3b. With increasing time degradation, RhB was degraded maximally, and maximum degradation was achieved in 9 min. The kinetic study was also investigated, and the plotted *C/C*_0_ first-order kinetic model (Figure 3c) result confirmed that **1** has a good degradation and adsorption ability against the RhB dye. The dye degradation percentages and the related *k* and *R* values are further calculated in Table 1. Therefore, for further experiments and a confirmation of our materials, we selected the RhB dye as a model and proceeded with experiments with it, as seen below.

**Figure 4:**
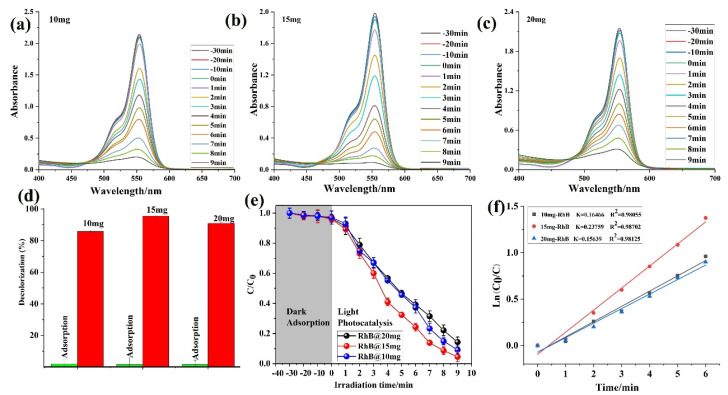
RhB concentration in mg: (**a**) 10 mg (**b**) 15 mg, (**c**) 20 mg; (**d**) adsorption percentage; (**e**) light and dark reaction; and (**f**) kinetic study.

The adsorption ability of **1** was investigated against the RhB dye in different milligram dye concentrations. In various mg concentrations (10, 15, and 20 mg), the ability of **1** was almost the same as shown in Figure 4a–c. Further, the comparison adsorption studies with the color solution were also examined. The capability was almost the same for all the three mg concentrations (Figure 4d). The photocatalytic ability was also tested in dark and light conditions. Still, there was not much effect, as shown in Figure 4e. The kinetic study was also plotted as a *C/C*_0_ first-order kinetic model (Figure 4f), and the result demonstrated that **1** shows a good degradation ability against the RhB dye. Moreover, the degradation percentages (%) (RhB mg concentration and kinetic study) were also calculated as shown in Table 2. The rate of Rh B photodegradation improved over an interval of 9 min at a certain catalyst concentration (15 mg). The activity was decreased as the catalyst concentration increased to 15 mg; this may be caused by the impacts of screening and light scattering. The result is very good, and **1** has an excellent photocatalytic ability [47].

**Figure 5:**
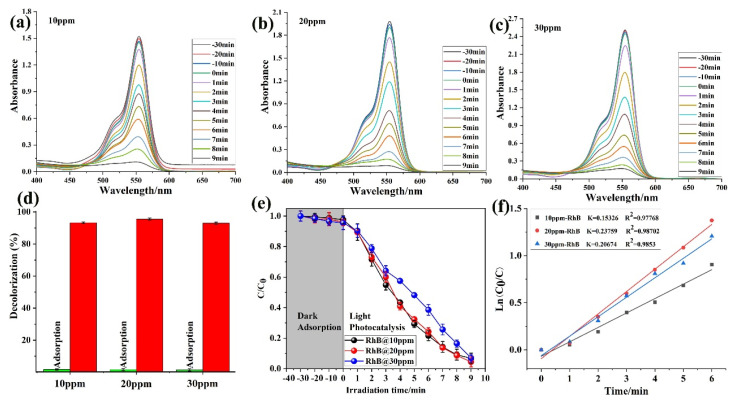
RhB concentration in ppm: (**a**) 10 ppm (**b**) 20 ppm, (**c**) 30 ppm; (**d**) adsorption percentage (the red color is degradation efficiency); (**e**) light and dark reaction; and (**f**) kinetic study.

The adsorption ability of **1** was investigated against the RhB dye in different ppm concentrations of solutions. In various ppm concentrations (10, 20, and 30 ppm) the ability of **1** was almost the same as shown in Figure 5a–c. Further, the comparison adsorption studies with color solutions were also examined. The capability was almost the same for all three ppm solutions (Figure 5d). The photocatalytic ability was also tested in dark and light conditions. Still, there was not much effect, as shown in Figure 5e. The kinetic study was also plotted as a *C/C*_0_ first-order kinetic model (Figure 5f). The result demonstrated that **1** shows a good degradation and adsorption ability against the RhB dye. Moreover, degradation percentages (%) (RhB ppm concentration and kinetic) were also calculated as shown in Table 3. Overall, the result is very good, and **1** has an excellent adsorption and photocatalytic ability. The presence of a greater concentration gradient of the Rh B dye, which enhances the diffusive contribution of the mass transfer process, can explain the increase in degradation in the experiments which had higher beginning concentrations of Rh B dye. The low sorption efficiency may be caused by the fact that at higher Rh B dye concentrations, there are fewer active sites on the surface of the structure than there are Rh B dye molecules available. As a result, sorbate molecules must compete with one another for the limited number of sorption sites on the surface of the material.

**Figure 6:**
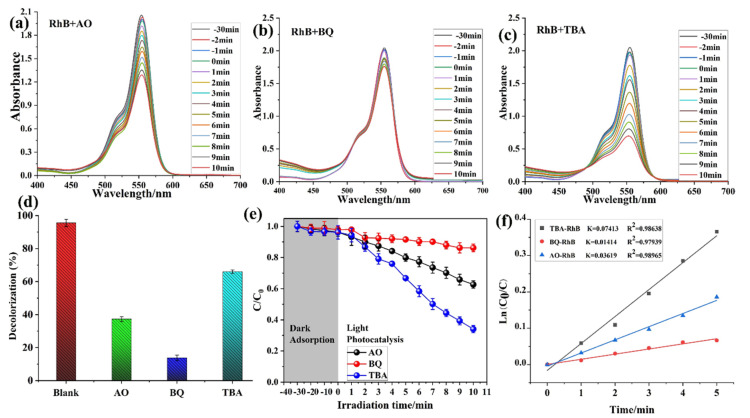
Active substance capture experiment in the process of RhB degradation: (**a**) RhB-AO, (**b**) RhB-BQ, and (**c**) RhB-TBA; (**d**) decolorization; (**e**) light and dark reaction of active substance; and (**f**) kinetic study.

**Figure 7:**
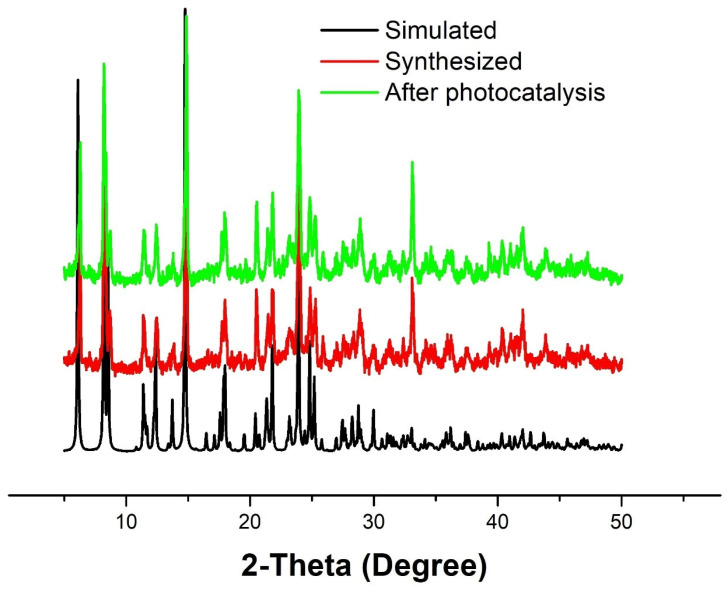
XRD spectra of simulated (black) synthesized CP 1 (red) and after photocatalysts (green).

Moreover, the active substance capture experiment was performed against RhB with different active substances such as AO, BQ, and TBA, as shown in Figure 6a–c. It is obvious from the investigation that the CP **1** photocatalyst is more active against RhB in the BQ species. Figure 6d shows us the comparison study of active species with a blank solution, and the result shows us that during the BQ active substance experiment, the CP **1** photocatalyst showed good activity. Furthermore, the dark and light reaction were also investigated against CP **1** and the active substance. The concentration ratios of MB, MO, MV, and RhB (*C*/*C*_0_) versus the time were plotted as shown in Figure 6e. As can be seen, the *C/C*_0_ values are as same in the dark reaction; meanwhile, in the light reaction, the BQ substances are more active and showed significant photocatalytic activity against the dye. In contrast, the others’ active substance activity decreased with the increasing reaction time in the presence of a catalyst, while the *C/C*_0_ values decreased very slowly in the light experiment. Moreover, a first-order kinetic equation for the active substance was plotted to describe the photocatalytic degradation kinetics of CP **1**. It was found that the active substance TBA-RhB has a *k* value of 0.07413, BQ-RhB has a *k* value of 0.01414, and AO-RhB has a *k* value of 0.03619, which shows us that CP **1** is better active in the presence of BQ (Figure 6f). Overall, the results are significant, and CP **1** showed a tremendous photocatalytic activity against dyes. The PXRD patterns of **1** before and after the photocatalytic reaction were examined. Due to the stability of **1** during the photocatalytic process, there were no noticeable changes in the morphology of **1** after four further runs (Figure 7). All the data are collected in Table 4, including the percentage (%) composition and kinetic calculation value of *k* and *R*^2^ pseudo-first-order kinetic study.

## 3. Materials and Method

### 3.1. Materials

The chemicals and reagents utilized in this study were of analytical grade and were not purified before use. Zinc acetate was bought from Syngenta, and the 2,5-bis(pyrid-4-yl)pyridine and 4-nitrobenzoic acid ligands were bought from Wanhua Chemical Company. The PXRD results were obtained using Cu-K radiation with a Bruker ADVANCE X-ray diffractometer (λ = 1.5418 Å). The Nicolet Impact 750 FTIR spectrometer was used to conduct the FTIR spectroscopy on the KBr disc. TGA measurements were performed from 25 to 900 °C at a heating rate of 10 °C/min in a nitrogen environment. The UV-Vis 2501PC spectrophotometer from Shimadzu was used to research the photocatalytic characteristics.

### 3.2. Preparation Methodology

A mixture of (bpyp = 2,5-bis(pyrid-4-yl)pyridine) (bpyp) (23.3 mg, 0.10 mmol), HL-NO_2_ (33.4 mg, 0.20 mmol), and Cd(CH_3_COO)_2_·2H_2_O (34.0 mg, 0.15 mmol) were dissolved in DMF/H_2_O (3 mL) in a screw-capped vial. After two drops of HNO_3_ (62%, aq.) were added to the mixture, the vial was capped and placed in an oven at 110 °C for 96 h. The resulting single crystals were washed with DMF and H_2_O and dried in air to give **1**. IR (KBr pellet, cm^−1^) spectra were as follows: 3037 (w); 2792 (w); 1945 (w); 1690 (m); 1605 (v); 1531 (v); 1346 (m); 1271 (v); 1105 (m); 987 (v); 864 (v); and 718 (m). Elemental analysis calCd(%) for C_29_H_19_CdN_5_O_8_ was as follows: C, 51.34; H, 2.80; and N, 10.33. We found the following: C, 53.06; H, 2.97; and N, 10.84% (ESI Figure S1).

### 3.3. Photocatalysis Study

The photocatalytic properties of **1** (40 mg) were assessed through the degradation of dye pollutants in an aqueous solution under a UV-400 photochemical reactor equipped with a 100 W mercury lamp (with a mean wavelength of 365 nm). A photocatalyst of 0.01 mmol was combined with an aqueous solution containing 50 mL of antibiotics at a concentration of 20 mg/L. The suspension comprising antibiotics and photocatalyst was subjected to magnetic stirring for 30 min under dark conditions until the establishment of adsorption–desorption equilibrium. Samples of 3.5 mL were extracted at 5 min intervals using a 3 mL pipette. The residual catalyst was removed through centrifugation to enable analysis via UV–visible spectroscopy spectrophotometer. An absorption wavelength was applied to monitor the photocatalytic degradation. Furthermore, the initial experiment was conducted under the subsequent reaction conditions: (1) absence of photocatalyst during UV irradiation; (2) presence of photocatalyst during UV irradiation with the existence of 2 mL tert-butanol (*t*-BuOH); (3) substitution of 2 mL *tert*-butanol (*t*-BuOH) with 2 mL benzoquinone (BQ); and (4) substitution of TBA with 2 mL ammonium oxalate (AO). The definition of antibiotic degradation efficiency is as follows:

Degradation efficiency = (*C*_0_ − *C*)/*C*_0_ × 100%

where *C*_0_ (mg/L) is the initial concentration of dyes, and *C* (mg/L) is the concentration of dyes at reaction time *t* (min).

## 4. Conclusions

In summary, CP **1** was produced under hydrothermal conditions utilizing a mixed ligand approach. The characterization of CP **1** was conducted using various analytical techniques, including SEM, FTIR, XRD, BET, an elemental analysis, and a TGA. In addition, CP **1** was utilized to eliminate environmental contaminants, specifically dyes. The efficiency of removal was found to be noteworthy, and the process of adsorption proceeded at a rapid pace. The adsorption and photocatalytic degradation of dyes on substrate **1** was a multifaceted phenomenon governed by a combination of physical and chemical interactions. Though it was believed that electrostatic contacts play a significant role in this adsorption process, the exothermic character of adsorption made it impossible to disregard the presence of physical interactions. Concerning various adsorbents, the dyes’ ability to bind to one another was compared.

## Figures and Tables

**Table 1:** Dye degradation percentage (%) and kinetic calculation.

S.No	Dye Degradation (%)	*k*	*R* ^2^
MB	29.24%	0.03614	0.98085
MO	35.44%	0.04192	0.97807
MV	58.92%	0.09838	0.9964
Rh B	95.52%	0.23759	0.98702

**Table 2:** Degradation kinetic rate (concentration used in mg).

	10 mg	15 mg	20 mg
Degradation (%)	90.87%	95.52%	85.76%
*k*	0.16466	0.23759	0.98125
*R* ^2^	0.98055	0.98702	0.98125

**Table 3:** Degradation percentage (%) (RhB ppm concentration and kinetic).

	10 ppm	20 ppm	30 ppm
Degradation (%)	92.99%	95.52%	93.24%
*k*	0.15326	0.23759	0.20674
*R* ^2^	0.97768	0.98702	0.9853

**Table 4:** Active substance degradation ability and kinetic study.

Active Substances	AO	BQ	TBA
Degradation (%)	37.29%	13.82%	65.98%
*k*	0.03619	0.01414	0.07413
*R* ^2^	0.98965	0.97939	0.98638

## Data Availability

Not applicable.

## Supplementary Materials

The following supporting information can be downloaded at: https://www.mdpi.com/article/10.3390/molecules28196848/s1, Figure S1. FTIR spectrum for **1**. Figure S2. TGA plot for **1**. Figure S3 View of the SEM before and after photocatalysis for sample **1**. Figure S4 Optical band gap of CP **1**. Scheme S1. view of the different coordination modes of nba in this work. Table S1. Selected crystallographic data for **1**. Table S2. Selected bond distances (Å) and angles (deg) for **1**.
