# Supplementary material for: A New Cd(II)-Based Coordination Polymer for Efficient Photocatalytic Removal of Organic Dyes
Source: Molecules. 2023 Sep 28;28(19):6848. doi: 10.3390/molecules28196848 (PMC10574129; doi:10.3390/molecules28196848)
Supplement: Supplementary file 1 [file molecules-28-06848-s001.zip › molecules-2600657-supplementary.pdf]

A new Cd(II)-based coordination polymer for efficient photocatalytic removal of organic dyes

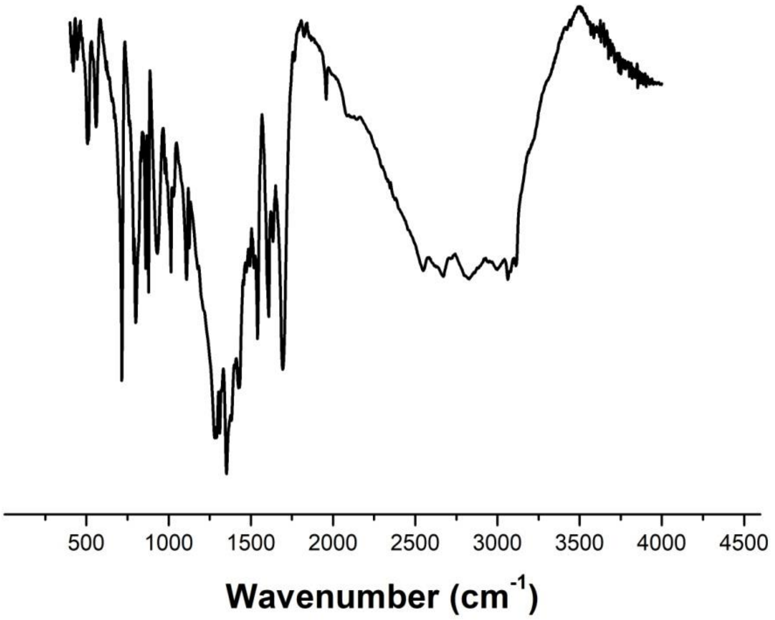

Fig. S1. FTIR spectrum for **1.**

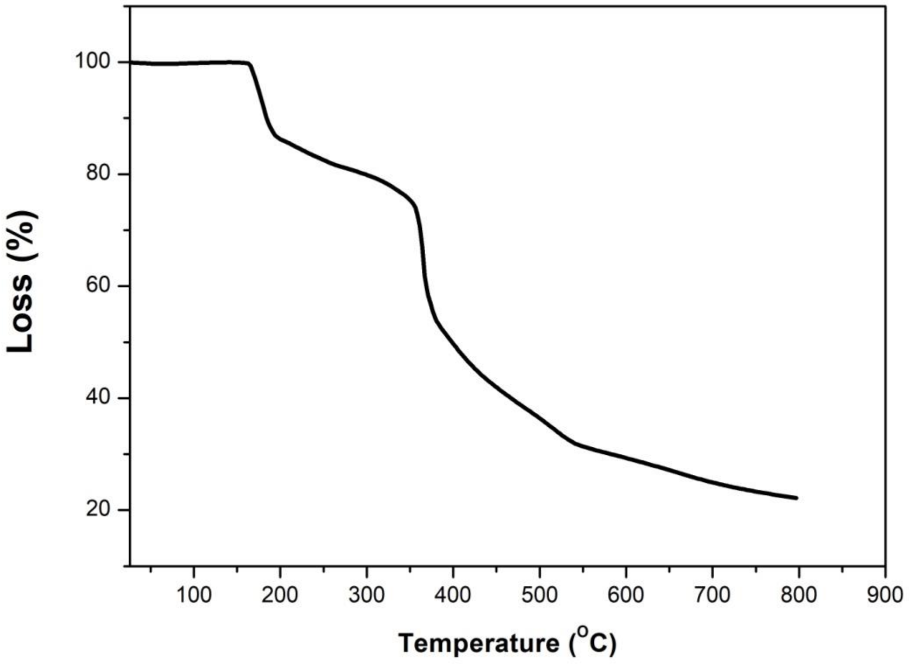

Fig. S2. TGA plot for **1**.

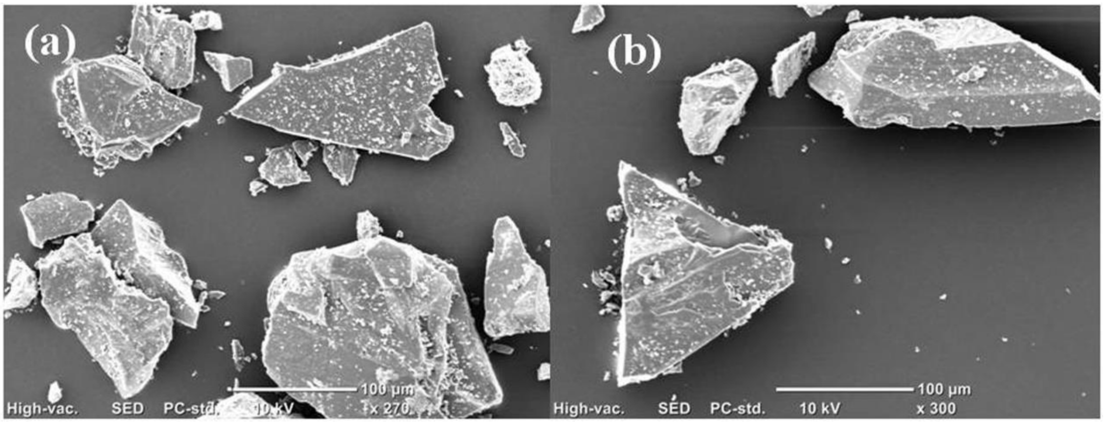

Fig. S3 View of the SEM before and after photocatalysis for sample **1**.

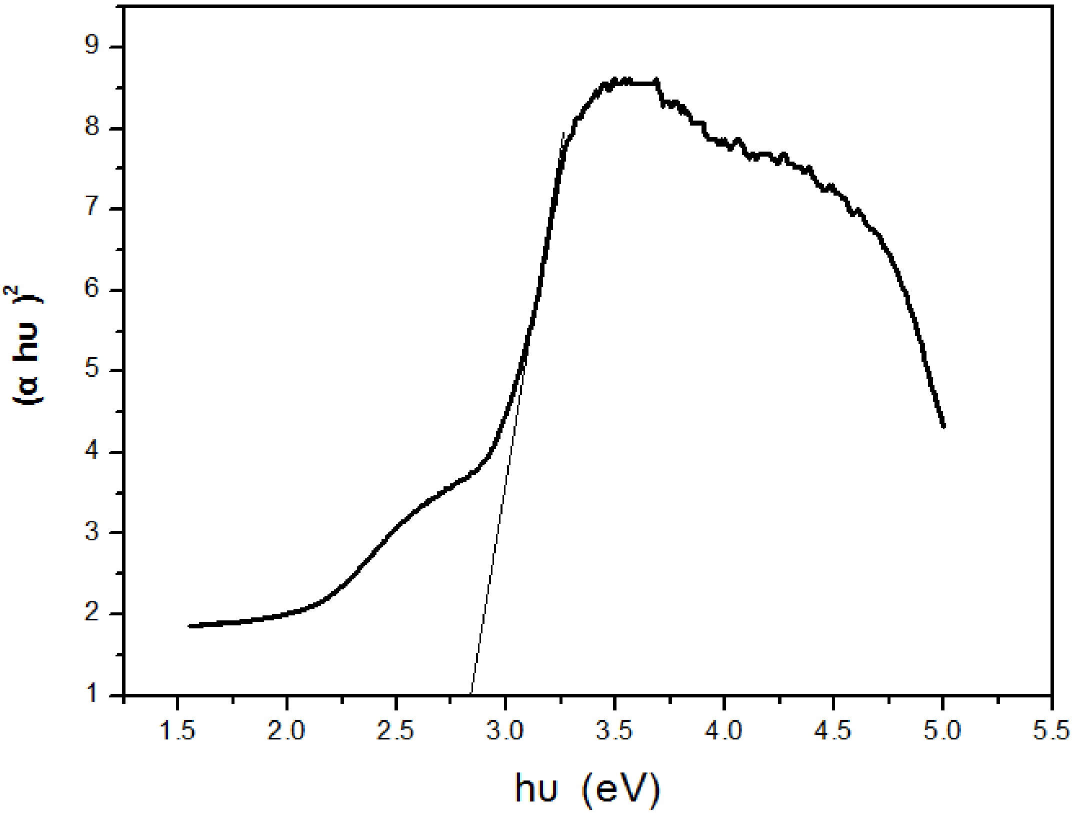

Fig. S4 Optical band gap of CP **1**.

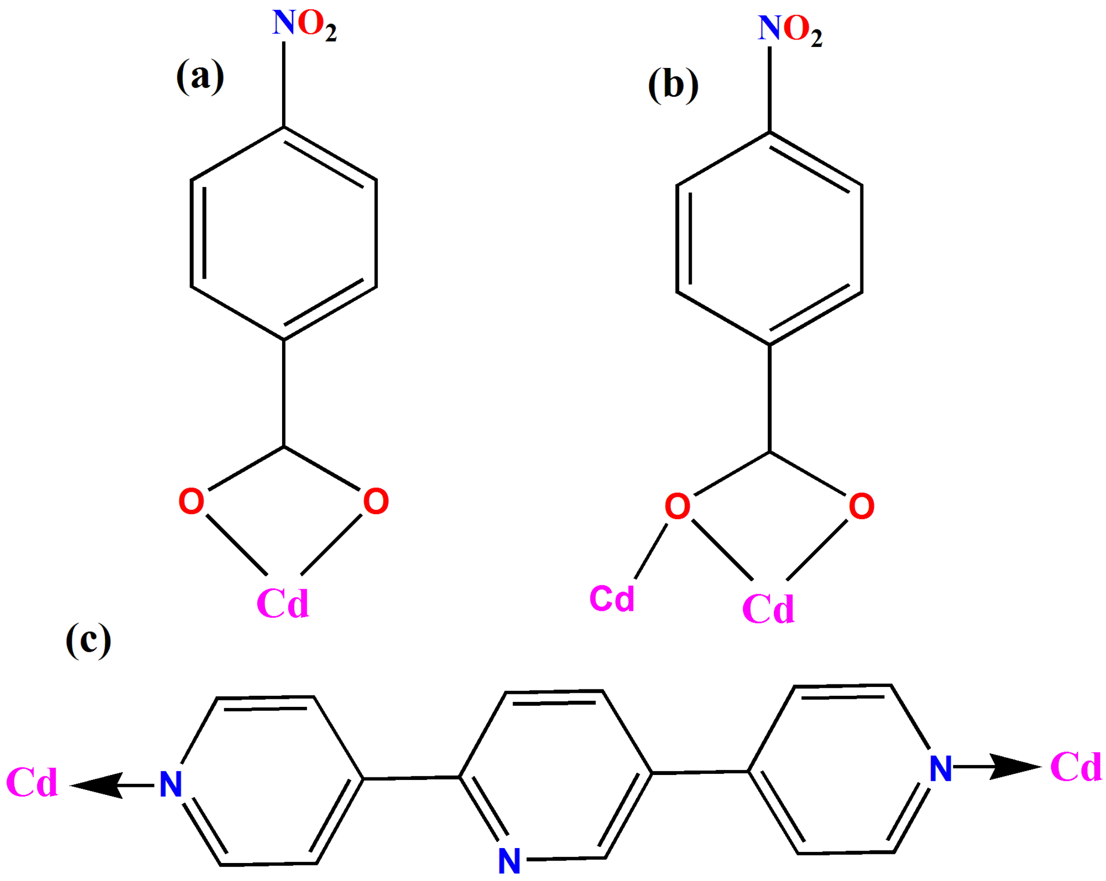

Scheme S1 view of the different coordination modes of nba in this work.

Table S1. Selected crystallographic data for **1**

Compound	**1**
Empirical formula	$C_{29}H_{19}CdN_5O_8$
Formula mass	677.89
Crystal system	Triclinic
Space group	P -1
a [Å]	8.6129(4)
b [Å]	11.7556(6)
c [Å]	15.8302(8)
α/(°)	68.709(1)
β/(°)	74.765(2)
γ/(°)	74.915(2)
V [Å^3]	1416.50(12)

Z	2
Tmin, Tmax	0.487,0.610
μ [mm^{-1}]	0.831
F [000]	680.0
Nref	4975
Final $R^{[a]}$ indices [I>2σ(I)]	R1 = 0.0396<br>wR2 = 0.0768

[a] $R_1=\sum||F_o|-|F_c||/\sum|F_o|$, $wR_2=[\sum w(F_o^2- F_c^2)^2/ \sum w(F_o^2)^2]^{1/2}$

Table S2. Selected bond distances (Å) and angles (deg) for **1**

**1**			
Cd(1)-O(3)	2.306(2)	Cd(1)-O(5)	2.443(2)
Cd(1)-O(6)	2.354(2)	Cd(1)-N(1)	2.310(3)
Cd(1)-N(3)	2.294(3)		
N(3)-Cd(1)-O(3)	86.48(9)	N(3)-Cd(1)-N(1)	173.78(10)
O(3)-Cd(1)-N(1)	87.32(9)	N(3)-Cd(1)-O(6)	99.13(9)
O(3)-Cd(1)-O(6)	144.41(9)	N(1)-Cd(1)-O(6)	86.28(9)
N(3)-Cd(1)-O(5)	94.58(10)	O(3)-Cd(1)-O(5)	90.38(9)
N(1)-Cd(1)-O(5)	86.07(10)	O(6)-Cd(1)-O(5)	54.28(8)
